# Draft Genome Sequence of *Meiothermus ruber* H328, Which Degrades Chicken Feathers, and Identification of Proteases and Peptidases Responsible for Degradation

**DOI:** 10.1128/genomeA.00176-13

**Published:** 2013-05-02

**Authors:** Shuhei Inada, Kunihiko Watanabe

**Affiliations:** Division of Applied Life Sciences, Graduate School of Life and Environmental Sciences, Kyoto Prefectural University, Shimogamo, Kyoto, Japan

## Abstract

*Meiothermus ruber* H328 was isolated from Arima Hot Springs, Kobe, Japan, as a moderate thermophile. It has a strong ability to degrade intact chicken feathers. The enzymatic mechanism of the strain for feather degradation is unclear. The draft genome suggests potent enzyme candidates for degradation of keratin, a hard-to-degrade protein found in feathers.

## GENOME ANNOUNCEMENT

*Meiothermus ruber* was first characterized as a species of the genus *Thermus* because of its production of an intracellular bright red or orange carotenoid (1). Then, the species was reclassified in the genus *Meiothermus* due to lower optimum growth temperatures than those for *Thermus* (2). *M. ruber* H328 was reported to have remarkable keratinolytic activity toward intact chicken feathers and was shown to be applicable for feather decomposition in combination with an acidulocomposting garbage-treatment process (3, 4). The keratinolytic proteases secreted by strain H328 to extracellular space are expected to play an important role in feather degradation. In our previous study, it was revealed that a single form of protease is not responsible for keratin degradation, and a complex form of keratinolytic proteases that were sensitive to serine protease inhibitors show extremely high stability against surfactants and organic solvents (unpublished data). However, we have not yet succeeded in completely purifying and characterizing them. Thus, we performed genome analysis of strain H328 as an alternative approach to investigating proteases and peptidases involved in feather degradation.

To determine the complete nucleotide sequence of the genome, chromosomal DNA was isolated from strain H328 as described previously (5), and the genome sequencing was performed by an Illumina GAIIx with a paired-end library (15,025,420 reads). The resulting reads were assembled by Velvet v1.0.12 (6), and 316 contigs were obtained (average length, 10,117 bp). The *N*_50_ contig length was 59,755 bp. The H328 draft genome was first constructed in 3.0 Mbp with coverage of 98.7% to the complete genome of *M. ruber* strain DSM 1279^T^ (7) by using BLASTx. Since 55 gaps occurred in the genome, gap fillings were performed by primer walking of PCR products using a capillary DNA sequencer. Finally, 53 of the 55 gaps were filled. The genes coding for proteins in this H328 draft genome were predicted based on similarity to the NCBI database using the software BLASTp with default cutoff values of *E* < 1 and with the low-complexity filter disabled.

The H328 draft genome was constructed as a single chromosome (3,029,288 bp, 63.5% G+C content). A total of 2,945 protein-coding genes and 53 RNA genes with high similarity to other bacterial genes in the H328 draft genome were identified, whereas 122 protein-coding genes were deleted compared with the genome of strain DSM 1279^T^. Seventy-eight candidate genes for protease and peptidase were found overall in the draft genome. The subcellular localization of each candidate was predicted by using LocateP (http://www.cmbi.ru.nl/locatep-db/cgi-bin/locatepdb.py), PSORTb (http://www.psort.org/psortb/), and CELLO (http://cello.life.nctu.edu.tw/cello.html). Then, putative signal peptides and promoter regions were predicted using SignalP v3.0 (http://www.cbs.dtu.dk/services/SignalP/) and GENETYX-MAC v10 (Genetyx Co., Tokyo, Japan), respectively. As a result, 26 protease and peptidase genes most probably involved in feather degradation were screened. Furthermore, 18 protease and peptidase genes that belong to a family of serine proteases were found. This result supports the fact that the keratinolytic activity was completely inhibited by serine protease inhibitors. As a next step, it is possible to investigate whether those gene products are involved in feather degradation.

### Nucleotide sequence accession numbers.

The draft genome sequence of *M. ruber* H328 was deposited in the DDBJ/EMBL/GenBank database under accession no. BAOR01000001 to BAOR01000004 and DF236949.
